# Interpretable Machine Learning with Prediction Uncertainty Quantification for *d*_33_ in (K_0.5_Na_0.5_) NbO_3_-Based Lead-Free Piezoelectric Ceramics

**DOI:** 10.3390/ma19050948

**Published:** 2026-02-28

**Authors:** Xiaohui Yuan, Yalong Liang, Bang Lu, Gaochao Zhao, Pei Li

**Affiliations:** 1 College of Architecture and Civil Engineering, Xinyang Normal University, Xinyang 464000, China; yxh@xynu.edu.cn (X.Y.); 18236071089@163.com (Y.L.); 2 Henan International Joint Laboratory of Structural Mechanics and Computational Simulation, College of Architectural and Civil Engineering, Huanghuai University, Zhumadian 463000, China; lb19515611979@163.com (B.L.); gczhao@mail.ustc.edu.cn (G.Z.); 3 Centre for Industrial Mechanics, Institute of Mechanical and Electrical Engineering, University of Southern Denmark, 6400 Sønderborg, Denmark

**Keywords:** KNN-based piezoceramics, interpretable machine learning, uncertainty quantification, SHAP analysis, SISSO descriptor

## Abstract

The accelerated discovery of high-performance lead-free piezoelectric ceramics is hindered by the vast compositional space and the limited interpretability of conventional machine learning (ML) models. Here, we propose a physics-informed and interpretable ML framework with integrated uncertainty quantification to predict and understand the piezoelectric coefficient *d*_33_ of (K_0.5_Na_0.5_) NbO_3_ (KNN)-based ceramics. A curated dataset of 1113 experimental samples is used to construct 65 descriptors by decoupling A-site and B-site ionic contributions. Pearson correlation analysis reduces these to an optimized 11-dimensional feature set for training deep neural networks, Wide & Deep networks, and residual networks. A Bayesian neural network further provides predictive uncertainty, which quantitatively reflects the confidence of machine-learning-based *d*_33_ predictions rather than experimental measurement uncertainty. To achieve physical interpretability, SHapley Additive exPlanations (SHAP) are combined with the Sure Independence Screening and Sparsifying Operator (SISSO) to derive a compact analytical descriptor revealing that sintering temperature, B-site electronic anisotropy, and A-site ionic displacement jointly govern *d*_33_. The proposed framework achieves high accuracy (R^2^ ≈ 0.81) while offering transparent design rules for next-generation lead-free piezoelectrics.

## 1. Introduction

High-performance piezoelectric ceramics constitute a unique class of functional materials, with (PbZr_1_ − xTixO_3_, PZT)-based ceramics being widely adopted in modern electronics due to their outstanding piezoelectric coefficients (*d*_33_ ≈ 200–1500 pC/N) and high Curie temperatures (Tc < 90–400 °C) [1]. However, increasing concerns over lead-related environmental and health risks have driven intensive efforts to develop sustainable lead-free alternatives [2,3,4,5,6]. In addition to oxide perovskites, emerging lead-free systems such as AlN- and AlScN-based materials have demonstrated promising ferroelectric and MEMS-compatible performance. While these nitride systems represent an important parallel development, the present study concentrates on bulk KNN-based ceramics, whose compositional richness and extensive experimental reporting make them particularly suitable for interpretable data-driven modeling [7,8]. Among the various candidates, [(K, Na) NbO_3_, KNN]-based ceramics stand out as one of the most promising alternatives due to their high *d*_33_ values and excellent temperature stability [9,10,11,12,13]. Although phase-boundary engineering has enabled *d*_33_ values approaching 550 pC/N in optimized KNN systems, the vast compositional and processing parameter space renders conventional trial-and-error approaches inefficient, highlighting the need for rapid and effective evaluation strategies.

The integration of machine learning (ML) with materials informatics has provided powerful tools for predicting and optimizing material properties and has been successfully applied to piezoelectrics, crystal structure prediction, ferroelectrics, and perovskite stability assessment [14,15,16,17,18,19]. However, the limited interpretability of ML models remains a major obstacle to their broader adoption in materials science [20,21,22,23,24]. To address this challenge, explainable artificial intelligence (XAI) has emerged to enhance model transparency by elucidating how complex learning models, such as deep neural networks, map input features to predicted properties [25,26,27].

In recent years, a variety of novel XAI techniques have been proposed, which can generally be categorized into global and local interpretation methods [28,29,30,31]. These can be grouped into global and local explanation methods. While global explanation methods interpret the decision-making of DNNs across a population by visualizing the signals that cause significant activation in a neuron [32,33], analyzing relationships between neurons [20] or by linking the neuron’s learned concepts to human-understandable concepts [34,35,36,37]. In contrast, local explanations provide interpretations of the prediction for a particular data example by attributing relevance to the input features [38,39].

Fundamentally, the interpretability of machine learning (ML) refers to the ability to examine and understand how input features are mapped to outputs in a mathematical and/or logical manner [1]. The ultimate goal of interpretability research is to develop model architectures that are inherently interpretable, thereby avoiding the black-box nature commonly associated with complex learning models [40].

In machine learning models, an excessive number of features can lead to the curse of dimensionality, increasing computational cost and causing model overfitting. In contrast, previous studies on perovskite materials have typically employed only a small number of features, often on the order of single digits [41,42,43,44,45]. Hu et al. [42] applied compositional and crystal-structure-related features to predict the piezoelectric coefficients of inorganic materials and screened 20 high-performance piezoelectric candidates from a dataset of 12,680 materials. He et al. [43] combined several feature selection techniques—namely Pearson correlation coefficient filtering, exhaustive feature search, and domain knowledge—to identify key descriptors governing the piezoelectric coefficient $d_{33}$ of BaTiO_3_-based ceramics. In this context, the sure independence screening and sparsifying operator (SISSO) has emerged as an effective interpretable ML approach, capable of deriving compact and explicit mathematical descriptors that are linearly correlated with target properties [46,47,48,49].

In this work, we develop an interpretable *d*_33_ descriptor construction framework that integrates physics-informed feature engineering, machine learning regression, Bayesian uncertainty quantification, and symbolic descriptor discovery to elucidate the composition–structure–property mapping of KNN-based ceramics across a broad compositional space. Here, uncertainty quantification specifically refers to the estimation of prediction uncertainty arising from model parameters and data variability, rather than experimental measurement fluctuations.

We deliberately focus on *d*_33_ because it is the most widely reported and standardized performance metric for bulk KNN ceramics, providing the most statistically consistent dataset for reliable model training and uncertainty-aware modeling.

The remainder of this paper progressively develops the proposed hybrid learning pipeline: Section 2 describes dataset construction and model design; Section 3 evaluates predictive performance across neural architectures, followed by Bayesian uncertainty analysis and SHAP–SISSO-based symbolic regression. Through this integration, the framework advances from accurate prediction to physically interpretable descriptor discovery, transforming a data-driven model into a transparent materials design tool.

## 2. Materials and Methods

### 2.1. Feature Selection

**Figure 1:**
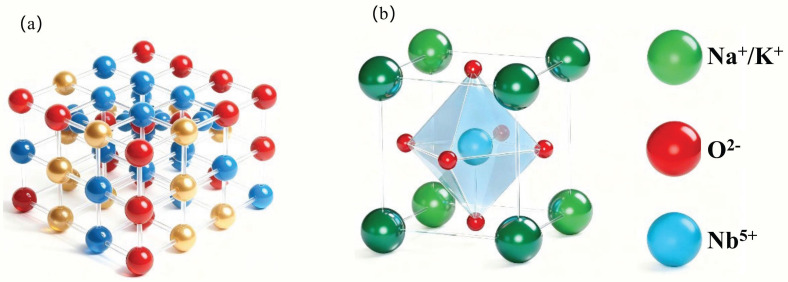
(**a**) Schematic diagram of the crystal structure of KNN-based materials. (**b**) Schematic diagram of the perovskite oxygen octahedral structure.

The KNN material, with the chemical formula (K_0.5_Na_0.5_) NbO_3_, is a perovskite-structured ceramic based on potassium, sodium, and niobium. At room temperature, its crystal structure typically exhibits an orthorhombic phase (Figure 1a). The piezoelectric properties of KNN-based ceramics originate from their characteristic ABO_3_-type perovskite crystal structure. As illustrated in Figure 1b, the fundamental framework of this structure consists of A-site ions (K^+^, Na^+^) located at the eight corners of the cubic lattice; B-site ions (Nb^5+^) positioned at the body center; and oxygen ions (O^2−^) occupying the six face centers, collectively forming [NbO_6_] oxygen octahedra centered around the B-site ions. The uniqueness of this structure lies in the fact that its piezoelectric response is closely related to the displacement of ions within the lattice, the distortion of oxygen octahedra, and the switching behavior of dipoles under an external electric field. Therefore, analyzing the intrinsic physicochemical properties of A-site and B-site ions based on this crystal structure is key to constructing effective performance descriptors.

**Figure 2:**
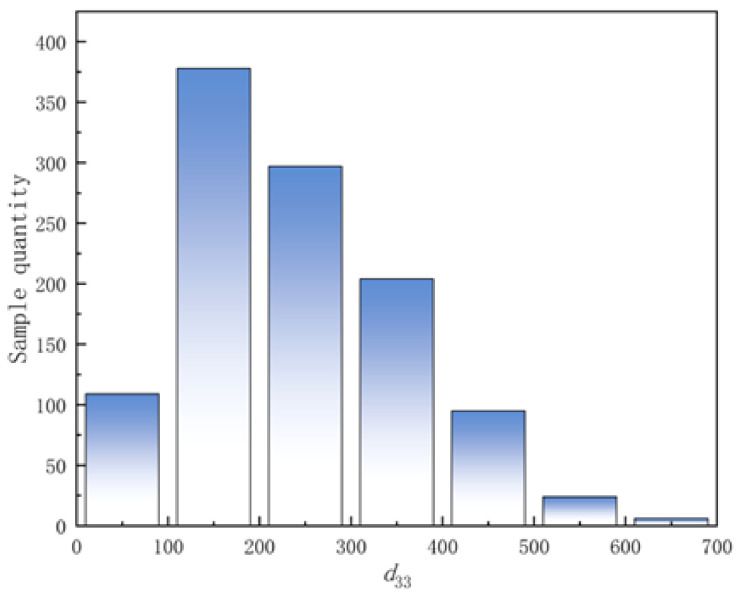
Statistical distribution of the $d_{33}$ dataset.

This study compiled 1113 experimental data points of KNN-based piezoelectric ceramics from 244 peer-reviewed publications. The corresponding data-source references are provided in the Supplementary References. The dataset was constructed through systematic screening of bulk KNN ceramics fabricated via conventional solid-state reaction to ensure comparability. Only samples with explicitly reported compositions, sintering conditions, and measured $d_{33}$ values were retained, with detailed references provided in the Supplementary Information. For identical compositions under comparable conditions, averaged values were adopted to reduce reporting bias. Records with incomplete processing details or ambiguous measurement information were excluded. All retained data were further subjected to outlier screening and normalization prior to model training. The dataset encompasses chemical compositions, sintering parameters, and corresponding piezoelectric coefficients ($d_{33}$) for each sample, with $d_{33}$ values distributed in the range of 0–700 pC/N as detailed in Figure 2. The comprehensive coverage of diverse compositional designs and processing conditions demonstrates favorable statistical distribution characteristics, establishing a reliable data foundation for subsequent modeling.

To overcome the inherent limitations of conventional “black-box” models, we adopted a first-principles-inspired framework to systematically elucidate the key physical parameters governing lattice distortion, electronic structure, and polarization mechanisms. By coupling symmetry breaking in ABO_3_ perovskite structures with the local chemical environment, we established a physics-informed heuristic strategy that enables independent representation of A-site and B-site cations. Multidimensional physicochemical descriptors were extracted separately for A-site and B-site ions, including electronegativity (Pauling scale), atomic radius, electron affinity, first ionization energy, nuclear magnetic moment, molar volume, ionic displacement, the ratio of valence electron number to nuclear charge (VEN/NC), polarizability, and lattice parameters. In addition, the tolerance factor (t) and octahedral factor (μ) were incorporated as key descriptors for evaluating perovskite structural stability, while the sintering temperature (ST) was introduced as a processing-related parameter. The resulting feature set comprised a total of 65 initial descriptors, comprehensively capturing both global structural characteristics and local chemical environment information, thereby providing a high-dimensional feature space for subsequent machine learning modeling.

### 2.2. Methodology

The proposed framework is inspired by first-principles physics, in which A-site and B-site ionic contributions in ABO_3_ perovskites are decoupled to construct 65 physically motivated compositional, structural, and electronic descriptors. Pearson correlation analysis was employed to reduce feature redundancy by retaining the most relevant descriptor from each highly correlated group.

Based on the selected descriptors, deterministic and probabilistic prediction models were developed using deep neural networks and Bayesian neural networks, enabling both accurate $d_{33}$ prediction and uncertainty quantification. Model interpretability was further enhanced through SHAP analysis and symbolic regression using the Sure Independence Screening and Sparsifying Operator (SISSO), yielding compact and physically interpretable descriptors.

**Figure 3:**
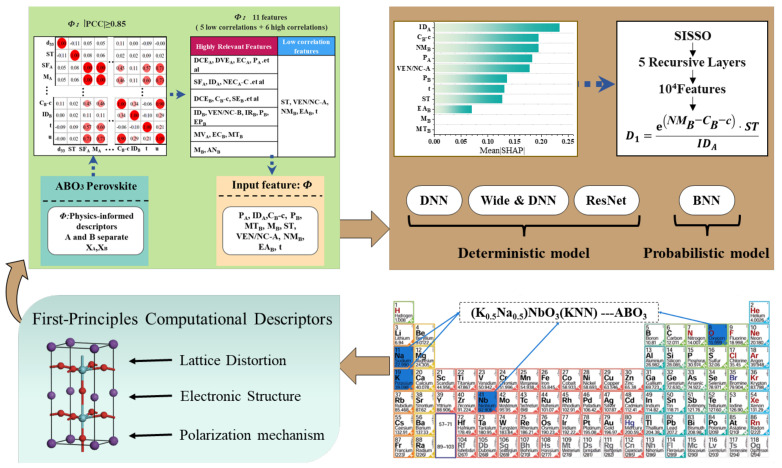
The physics-informed machine learning framework for the piezoelectric property prediction of ABO_3_ perovskites.

Overall, the framework integrates physics-informed feature construction, machine learning regression, uncertainty quantification, and interpretability analysis into a unified workflow, as illustrated in Figure 3.

#### 2.2.1. Data Preprocessing

During feature selection, Pearson correlation coefficients were calculated to quantify inter-feature correlations among the 65 initial descriptors,

(1) $$r_{ij}=\frac{cov(x_{i},x_{j})}{\sigma_{x_{i}}\sigma_{x_{j}}}$$

where $x_{i}$ and $x_{j}$ denote two feature variables. Based on the correlation matrix, descriptors were classified into highly correlated and weakly correlated groups (see Appendix A). From each highly correlated group, the feature exhibiting the strongest correlation with the piezoelectric coefficient $d_{33}$ was retained, while all weakly correlated features were preserved, resulting in an optimized set of 11 descriptors used as model inputs.

Data preprocessing was subsequently performed on the selected features. Outliers were identified and removed using the interquartile range (IQR) criterion, defined as

(2) $$IQR=Q_{3}-Q_{1}$$

with outlier thresholds given by

(3) $$Q_{1}-k\;IQR\leq x\leq Q_{3}+k\;IQR$$

where $Q_{1}$ and $Q_{3}$ represent the first and third quartiles, respectively. A conservative range of *k* = 2.5–3.0 was adopted to suppress extreme outliers while preserving experimental integrity.

To ensure stable convergence and effective training of the neural network models, a data-driven normalization strategy was adopted. Multiple normalization schemes were systematically evaluated, and based on comparative performance across six candidate methods, the final preprocessing primarily employed robust scaling, standard scaling, and power transformations using the Yeo–Johnson and Box–Cox methods (implemented via the PowerTransformer) [50].

Standard scaling transforms each feature according to

(4) $$x^{\prime }=\frac{x-\mu }{\sigma }$$

where μ and σ denote the mean and standard deviation of the feature, respectively. Robust scaling, which is less sensitive to outliers, is defined as

(5) $$x^{\prime }=\frac{x-\mathrm{median}(x)}{\mathrm{IQR}}$$

where IQR represents the interquartile range.

For features exhibiting strong skewness or non-Gaussian distributions, power transformations were applied. The Box–Cox transformation is expressed as

(6) $$x^{\prime }=\{\begin{array}{cc} \frac{x^{\lambda }-1}{\lambda }, & \lambda \neq 0\\ ln(x), & \lambda =0 \end{array}$$

while the Yeo–Johnson transformation extends this formulation to accommodate zero and negative values. These normalization strategies improve the distributional properties of the input features and enhance the robustness and generalization capability of the subsequent machine learning models.

#### 2.2.2. Neural Network Architecture Design

**Figure 4:**
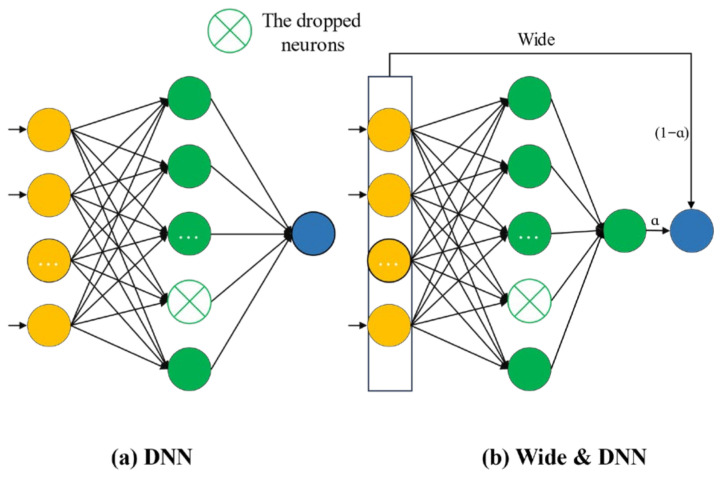

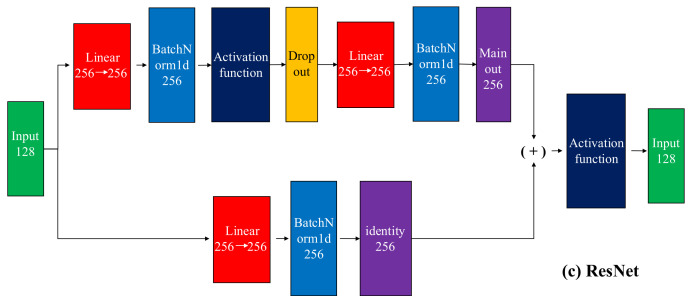
Schematic architecture of the proposed neural network models. (**a**) DNN with dropped neurons. (**b**) Wide & Deep architecture. (**c**) Detailed configuration of the ResNet-based residual.

The prediction of piezoelectric material properties is inherently challenging due to the high dimensionality of input descriptors, the strongly nonlinear relationships among variables, and the limited size and imbalanced distribution of available experimental datasets. Distinct neural network architectures differ substantially in their feature extraction mechanisms, gradient propagation stability, and levels of model interpretability. To systematically evaluate the representational capacity and generalization performance of different neural network architectures in the prediction of piezoelectric ceramic properties, this study constructs and comparatively analyzes three representative deep learning models, namely a conventional deep neural network (DNN), a Wide & Deep architecture [51,52], and a residual neural network (ResNet) [53,54], whose overall structural schematics are illustrated in Figure 4.

As shown in Figure 4a, linear models offer high parameter-level interpretability by explicitly describing additive relationships between input features and the response variable through direct linear mappings, but they are limited in capturing nonlinear interactions. The Wide & Deep architecture (Figure 4b) integrates a linear branch with a deep nonlinear branch, retaining explicit feature interpretability from the linear component while leveraging deep networks to model high-order interactions, thereby achieving a balance between interpretability and predictive performance. In contrast, ResNet (Figure 4c) enhances training stability and information propagation through residual connections, enabling effective modeling of complex nonlinear relationships. However, their interpretability is largely structural rather than parameter- or feature-level due to their hierarchical abstraction.

A comparative analysis of these architectures is therefore essential to elucidate the trade-offs between interpretability and modeling capability, as summarized in Table 1.

**Figure 5:**
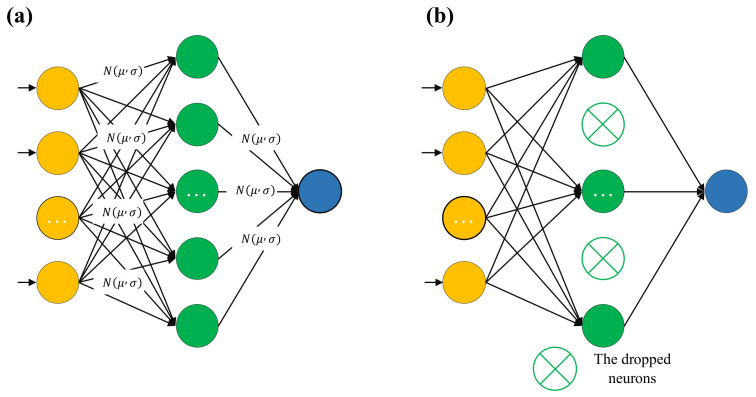
Schematic of two BNNs with (**a**) probabilistic weights and (**b**) probabilistic structure.

A Bayesian neural network (BNN) was developed to model the nonlinear relationship between material descriptors and piezoelectric properties. Unlike conventional neural networks, the BNN treats weights and biases as Gaussian-distributed probabilistic variables. Variational inference approximated the posterior by minimizing the Kullback–Leibler divergence, while the loss combined a mean-squared error term with KL regularization to balance accuracy and complexity. Monte Carlo sampling over the posterior provided predictive means and prediction uncertainty quantification. This probabilistic framework not only improves robustness but also enables uncertainty-aware interpretability, reflecting the relative importance and stability of input descriptors. Such capabilities are particularly valuable for piezoelectric ceramics, where data are limited, and material responses are highly nonlinear. The network architecture is shown in Figure 5.

As the study focuses on how network architectures and algorithms affect interpretability, detailed model derivations, hyperparameters, and implementation are provided in Appendix B.

The model’s generalization capability and robustness are rigorously evaluated using the following elements.

Coefficient of determination (R^2^): This measures the proportion of variance explained by the model (Equation (7)).

(7) $$R^{2}=1-\frac{{\sum_{i=1}^{n}\left(y_{i}-\overset{\wedge }{y_{i}}\right)}^{2}}{{\sum_{i=1}^{n}\left(y_{i}-\overset{-}{y}\right)}^{2}}$$

Root-mean-square error (RMSE): This quantifies absolute prediction errors (Equation (8)).

(8) $$RMSE=\sqrt{\frac{\sum_{i=1}^{n}{\left(y_{i}-{\overset{\wedge }{y}}_{i}\right)}^{2}}{n}}$$

Let $y_{i}$ denote the experimentally measured output value for the *i*-th sample, ${\overset{\wedge }{y}}_{i}$ represent the corresponding model-predicted value, and $\overset{-}{y}$ be the arithmetic mean of the observed dataset, where *n* is the total number of samples.

#### 2.2.3. Interpretability Framework: SHAP and SISSO

To quantify nonlinear and synergistic contributions of input features, the SHAP (Shapley Additive Explanations) framework was used. SHAP, based on cooperative game theory, interprets the model output as a total “payoff” distributed among features, providing a consistent measure of feature importance while accounting for interdependencies.

The Shapley value is mathematically expressed as

(9) $$\theta_{i}(f,x)=\sum_{r\in R}\frac{1}{\left|I\right|}\left[f_{x}\left(P_{i}^{r}\cup \{i\}\right)-f_{x}\left(P_{i}^{r}\right)\right]$$

where $\theta_{i}(f,x)$ denotes the Shapley value of the *i*-th feature for the input *x*; Rrepresents all possible permutations of the feature set, and ∣I∣ is the total number of features; $P_{i}^{r}$ corresponds to the subset of features that precede feature *i* in permutation r; and $f_{x}(\cdot )$ is the model prediction under the specified feature subset. This formulation allows SHAP to attribute prediction outcomes to individual features in a way that is both fair and mathematically rigorous.

Unlike SHAP, which quantifies the contribution of each input variable to the model output, the SISSO (Sure Independence Screening and Sparsifying Operator) framework aims to construct a compact, physically interpretable analytical descriptor that directly relates input features to the piezoelectric response. Rooted in compressed sensing theory, SISSO efficiently explores extremely large feature spaces formed by mathematically generated combinations of the original variables.

The SISSO procedure comprises two main steps. First, the Sure Independence Screening (SIS) evaluates the linear correlation between each candidate feature and the target variable $d_{33}$, retaining only the most relevant subset from the high-dimensional feature pool. Next, the Sparsifying Operator (SO) performs sparse regression on this reduced set to identify the optimal descriptor that achieves a balance between predictive accuracy and algebraic simplicity. The optimization process can be formalized as

(10) $$\underset{w}{min}{\left\|y-\sum_{i}w_{i}D_{i}\right\|}_{2}^{2}+\lambda ||w{||}_{1}$$

where $D_{i}$ denotes a candidate descriptor, w is the coefficient vector, and λ controls the sparsity level to prevent overparameterization.

SHAP was first used to identify features most relevant to $d_{33}$, narrowing the feature space. SISSO then performed multi-level symbolic feature expansion and iterative screening to derive a single low-dimensional descriptor capturing the combined effects of processing temperature, lattice distortion, and B-site electronic characteristics. Compared with black-box neural networks, the SISSO descriptor offers strong physical interpretability, low dimensionality, and direct applicability to material design, providing both accurate predictions and mechanistic insight. This SHAP → SISSO workflow ensures the descriptor is data-driven yet physically grounded.

## 3. Results

### 3.1. Performance Comparison Among the Three Architectures

**Figure 6:**
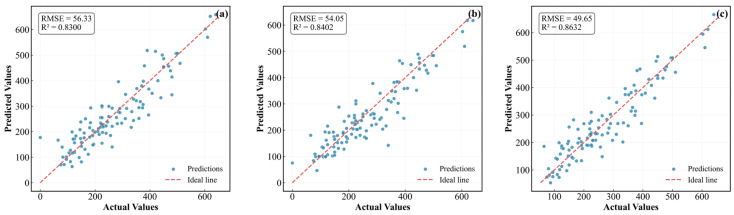
Regression plots of the experimental vs. predicted values. (**a**–**c**) represent DNN, Wide & DNN, and ResNet models, respectively. Performance metrics (RMSE and R2) are provided in the insets.

Figure 6 presents the regression results of the piezoelectric coefficient ($d_{33}$) between experimentally measured and model-predicted values for the three neural network models. Quantitative performance metrics, including the coefficient of determination (R^2^) and the root-mean-square error (RMSE), are summarized in each subplot.

Figure 6 summarizes the predictive performance of three representative neural network architectures trained on the optimized 11-dimensional physically relevant descriptor set for KNN-based piezoelectric ceramics. All models were trained and evaluated using identical data preprocessing, normalization, and partitioning schemes to ensure a fair and physically consistent comparison.

As shown by the clustering of data points around the parity line in Figure 6a–c, all three architectures can accurately reproduce the experimentally reported $d_{33}$ values extracted from the 244 peer-reviewed literature sources described in Section 2.1. This confirms that the descriptor design—incorporating A-site/B-site decoupling, lattice distortion metrics, electronic structure parameters, and processing conditions—effectively captures the dominant structure–property relationships governing the piezoelectric performance of KNN-based ceramics.

The models’ high predictive accuracy highlights an inherent trade-off between performance and interpretability (Table 2). While the ResNet architecture delivers the most precise deterministic predictions, its deeply nonlinear nature limits direct physical interpretability at the parameter level. To address this, Bayesian neural networks (BNNs) were employed for uncertainty quantification, and SHAP–SISSO analysis was used for post hoc interpretability and extraction of physically meaningful descriptors. Together, these components establish a unified framework that balances predictive accuracy, reliability, and physical insight.

### 3.2. Uncertainty Analysis Using Bayesian Models

Deterministic models perform point estimation, returning only a single predicted value while treating model parameters as fixed. For piezoelectric material systems, experimental data inherently contain systematic uncertainties arising from variations in synthesis conditions, microstructural heterogeneity, and instrumental measurement noise. Consequently, relying solely on a single deterministic prediction is insufficient to fully support materials design or experimental decision-making. A reliable predictive model should additionally quantify the confidence associated with its predictions.

The BNN represents network weights as probability distributions and is optimized via variational inference. During the prediction stage, the BNN performs multiple stochastic forward passes, enabling the estimation of both predictive mean and predictive uncertainty (expressed as the standard deviation). Although the BNN yields a slightly lower test accuracy (R^2^ = 0.77) compared to the deterministic ResNet model, it achieves a low mean uncertainty ratio of 8.60%, indicating that the predicted confidence intervals are narrow and statistically meaningful. The modest reduction in R^2^ originates from the KL regularization, which encourages smoother and more conservative predictions, thereby trading a small portion of accuracy for significantly enhanced reliability and generalization stability. By contrast, while ResNet attains a higher accuracy, it lacks the ability to express prediction confidence, limiting its interpretability in scientific and engineering applications.

Overall, a clear trade-off between predictive accuracy and reliability is observed. While ResNet achieves the highest deterministic accuracy, the BNN provides a more balanced and uncertainty-aware predictive framework, with an R^2^ of 0.77 and a low mean relative uncertainty of 8.60%. To further support experiment-oriented decision-making, we explicitly define a quantitative reliability criterion: predictions with a relative uncertainty exceeding 15%—approximately twice the mean uncertainty level—are considered potentially unreliable and should be treated with caution or excluded from experimental validation. This uncertainty-guided screening strategy enhances the interpretability and trustworthiness of the BNN, making it particularly suitable for piezoelectric material screening and performance evaluation.

**Figure 7:**
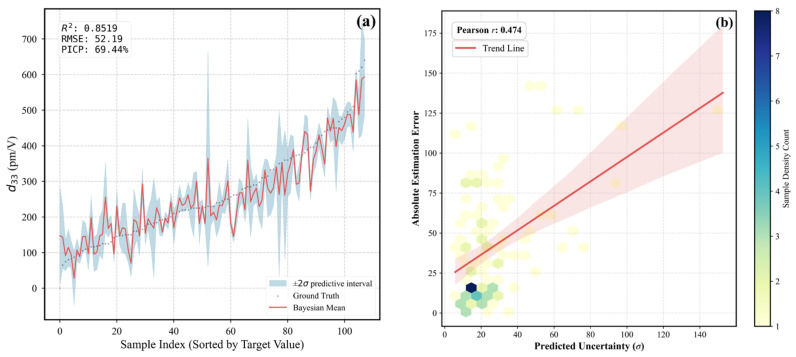
Bayesian prediction performance and uncertainty analysis for $d_{33}$. (**a**) Prediction results with uncertainty intervals. (**b**) Correlation between predictive uncertainty and estimation error. The red line indicates the regression fit with a 95% confidence interval (shaded).

Figure 7b shows the relationship between prediction error and predictive uncertainty obtained from the Bayesian neural network. Samples associated with larger prediction errors are generally accompanied by higher uncertainty, indicating that the model effectively identifies regions of reduced confidence. As discussed later, these high-uncertainty regions are closely related to increased physical complexity in the underlying structure–property relationships, which are further elucidated through SISSO-derived descriptors in Section 3.3.

### 3.3. Analysis Using SHAP and SISSO

**Figure 8:**
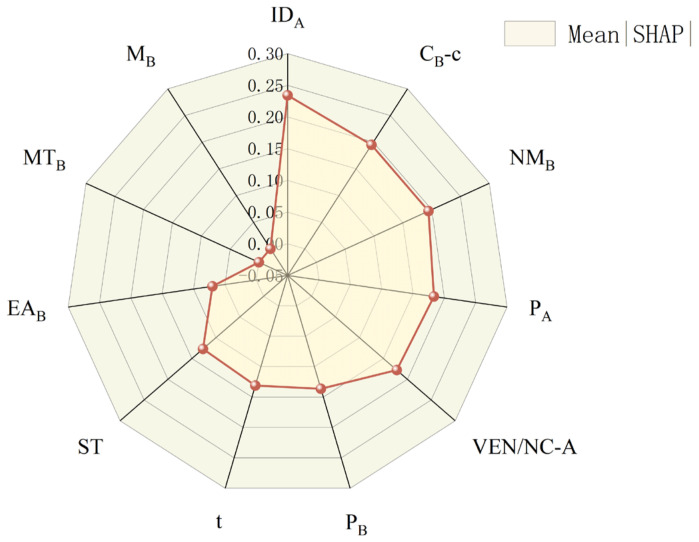
Global feature importance analysis of the ResNet model based on the mean absolute SHAP values.

Following the interpretability workflow described in Section 3.3, SHAP was first used to determine the relative contributions of each input descriptor to the prediction of $d_{33}$. The SHAP ranking showed that only a subset of descriptors exerted dominant influence, while features with negligible contributions were excluded. These retained key descriptors then served as the input basis for the subsequent SISSO process, ensuring that the descriptor construction was grounded in physically meaningful and model-relevant variables. Figure 8 shows the detailed results.

The preliminary feature importance analysis revealed that MT_B_ and M_B_ exhibit insignificant contributions to the model, indicating that these variables do not meaningfully influence the prediction of $d_{33}$. Consequently, both features were removed from the descriptor search space. The refined feature set was then subjected to the SISSO (Sure Independence Screening and Sparsifying Operator) methodology to identify physically interpretable, low-dimensional descriptors. The detailed definitions and physical interpretations of the retained features are presented in Table 3.

To construct an interpretable predictive model for $d_{33}$, the SISSO method was employed. SISSO integrates global feature screening (SIS) with sparsity-driven optimization (SO), enabling the discovery of meaningful nonlinear descriptors while maintaining a compact model structure and strong physical interpretability. Based on the nine primary physical features, a systematically expanded feature space was generated using the following mathematical operator set:

(11) $$\{+,\;-,\;\times ,\;÷,\;\exp,\;\log,\;\sqrt{\;},\;abs,\;\sin,\;\cos\}$$

A five-layer recursive feature expansion (feature depth = 5) was performed. In this procedure, the features generated at each layer were used as inputs for the subsequent layer, resulting in a progressively nested nonlinear feature construction. As the recursive depth increases, the size of the feature space grows exponentially. Thus, even when starting from only nine primary physical features, the five-layer expansion yields approximately 10^4^ candidate expressions. These candidates span a broad spectrum of functional forms, ranging from simple linear relationships to complex high-order coupling terms. An example is given below (Table 4).

This hierarchical feature construction process enables a comprehensive exploration of the descriptor space, ranging from simple monotonic relationships to complex nonlinear couplings involving electronic structure, crystallographic distortion, and processing conditions. In the SIS stage, the correlation between each candidate descriptor and the experimental $d_{33}$ values was evaluated, and only the top-ranked descriptors were retained for further consideration. This preliminary filtering significantly reduces the dimensionality of the search space while ensuring that physically meaningful features are preserved.

Subsequently, the sparsifying operator was applied in the SO stage to identify a concise expression that balances predictive accuracy and descriptor simplicity (see Equation (12)). The resulting optimal descriptor, denoted as D_1_, integrates the sintering temperature (ST), the electronic environment of the B-site cation (NM_B_-C_B_-c), and the A-site ionic displacement (ID_A_):

(12) $$D_{1}=\frac{e^{\left(NM_{B}-C_{B}-c\right)}\cdot ST}{ID_{A}}$$

**Figure 9:**
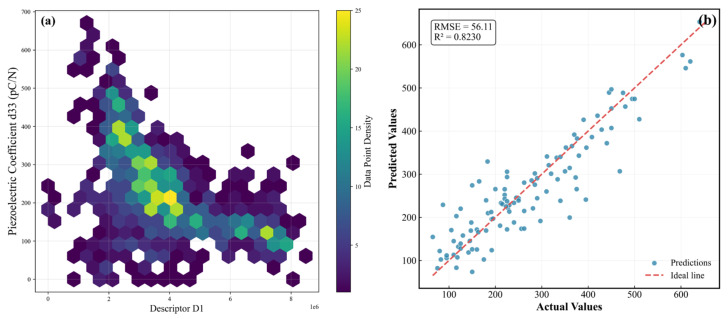
(**a**) Correlation between descriptor D_1_ and piezoelectric coefficient $d_{33}$. (**b**) Comparison between predicted and actual values for the ResNet model.

This descriptor highlights a coupled mechanism in which thermally activated densification, B-site electron–lattice interactions, and lattice distortion collectively govern the polarization response. To examine the physical validity of D_1_, its values were numerically computed and incorporated into an extended feature set for retraining the ResNet model. The resulting predictive performance retained high accuracy, with an R^2^ of 0.81 and an RMSE of 57.80 pC/N (see Figure 9) on the test dataset, confirming that D_1_ captures the dominant structure–property relationship rather than serving as an empirical fitting term.

A detailed physical interpretation of the descriptor further reinforces its significance. The sintering temperature (ST) regulates densification behavior, influencing grain size evolution, porosity elimination, and domain-wall mobility. The difference term (NM_B_-C_B_-c) reflects variations in B-site electronic anisotropy and lattice distortion along the c direction, which are associated with changes in Nb–O bond characteristics and the distortion of NbO_6_ octahedra in KNN-based perovskites. Such structural and electronic variations affect how easily the polarization direction can reorient within the lattice and may influence domain-wall motion. From a data-driven perspective, (NM_B_-C_B_-c) can therefore be regarded as an effective descriptor capturing the coupling between the B-site electronic environment and polarization switching behavior, rather than a direct microscopic measure of domain-wall energetics. Conversely, the A-site ionic displacement (ID_A_) reflects the degree of lattice distortion arising from cation off-centering and the associated polar instability. A smaller ID_A_ indicates greater structural compliance, enabling more facile polarization reorientation. Therefore, the ratio structure of D_1_ effectively expresses the balance between polarization mobility and lattice anchoring.

Furthermore, when visualized in a two-dimensional D1–$d_{33}$ map (see Figure 9), high-performance KNN compositions cluster in a well-defined region, demonstrating that the descriptor not only captures physical causation but also serves as a practical indicator for compositional optimization. This clustering trend indicates that compositions with favorable B-site electronic anisotropy and moderate lattice distortion consistently exhibit enhanced domain-wall mobility and polarization rotation freedom, which are key contributors to high $d_{33}$. Such clustering behavior suggests that D_1_ can be used prospectively to guide experimental synthesis by adjusting either the B-site electron density environment or the sintering thermal profile to achieve targeted electromechanical performance.

Overall, the integration of SHAP and SISSO yields a unified interpretation framework in which SHAP identifies the most influential variables, and SISSO constructs a concise analytical descriptor that quantitatively expresses how these variables jointly regulate $d_{33}$. This combined approach bridges data-driven learning and mechanistic understanding, allowing the prediction model to move beyond black-box inference toward physically grounded insight.

## 4. Conclusions

This work demonstrates a robust, physics-informed machine learning strategy for navigating the complex design space of KNN-based ceramics. Our findings indicate the following:

1. Model Performance: The Wide & Deep architecture outperforms standard networks by simultaneously processing low-dimensional physical descriptors and high-dimensional nonlinear features.
2. Predictive Reliability: The integration of Bayesian uncertainty quantification provides a confidence metric, which is essential for guiding experimentalists toward low-risk, high-reward compositions.
3. Physical Interpretability: The SHAP–SISSO pipeline successfully translated a “black-box” model into a human-understandable analytical descriptor (D_1_). This descriptor identifies that a B-site electronic structure and A-site displacement are the primary drivers of enhanced $d_{33}$.

In summary, this work establishes a general paradigm in which deep learning, uncertainty quantification, and symbolic regression are unified into a single interpretable framework. Beyond KNN-based ceramics, the proposed SHAP–SISSO strategy is readily transferable to other functional oxides, offering a practical route for data-driven yet physics-guided materials discovery.

Future work will extend this framework toward multi-property prediction and figure-of-merit optimization, incorporating additional piezoelectric and dielectric parameters as more consistently curated datasets become available. Furthermore, integrating active learning and closed-loop experimental validation may enable accelerated and uncertainty-aware material discovery.

## Figures and Tables

**Table 1:** Comparison of interpretability, advantages, and limitations of different neural network architectures.

Model Architecture	Interpretability	Advantages	Limitations
DNN	Medium	Strong nonlinear representation	Limited feature transparency
Wide & Deep	High	Interpretable linear component	Partial black-box behavior
Residual Network	Low	Stable training of deep models	Poor feature interpretability

**Table 2:** Comparison of predictive performance and interpretability among different neural network architectures.

Model Architecture	R^2^	RMSE (pC/N)	Interpretability Level
DNN	0.83	56.33	Medium
Wide & Deep	0.84	54.05	High
ResNet	0.86	49.65	Low

**Table 3:** SHAP analysis was performed on the ResNet-based model to derive the features and corresponding descriptions that impact the model’s weighting scheme.

Feature	Description
ST (°C)	Sintering temperature
VEN/NC-A	Ratio of valence electron number to nuclear charge of A-site
NM_B_	B-site Nuclear magnetic moments
EA_B_	Electron affinity of B-site element
t	Tolerance factor calculated by Shannon’s ionic radii
P_A_	Polarizability of A-site element
ID_A_	A-site Ionic displacement
P_B_	Polarizability of B-site element
C_B_-c	Cell parameters of B-site element in the c direction

**Table 4:** Hierarchical feature expansion and representative candidate expressions generated across recursive SISSO layers.

Hierarchy	Representative Expression
Layer 1	$e^{\left({\mathrm{NM}}_{B}{-C}_{B}-c\right)}$
Layer 2	$\frac{e^{\left({\mathrm{NM}}_{B}{-C}_{B}-c\right)}}{{\mathrm{ID}}_{A}^{2}}$
Layer 3	$\frac{e^{\left({\mathrm{NM}}_{B}{-C}_{B}-c\right)}\times \mathrm{ST}}{{\mathrm{ID}}_{A}^{2}}$
Layer 5	$\sin\left(\frac{e^{\left(\sin(\mathrm{VEN}/\mathrm{NC}-A)\right)}}{{|\mathrm{NM}}_{B}{/\mathrm{EA}}_{B}|}\right)$

## Data Availability

The original contributions presented in this study are included in the article and Supplementary Materials. Further inquiries can be directed to the corresponding author.

## Supplementary Materials

The following supporting information can be downloaded at https://www.mdpi.com/article/10.3390/ma19050948/s1: Experimentaldata.xlsx, This file contains the dataset used in this study (1113 experimental samples with 65 features) and the table of Supplementary References cited in the Supplementary Materials. References [55,56,57,58,59,60,61,62,63,64,65,66,67,68] are cited in the Supplementary Materials.

## Abbreviations

The following abbreviations are used in this manuscript:

**Feature**	**Description**
SF_A_	Atomic electron scattering factor at 0.5 of A-site element [55]
SF_B_	Atomic electron scattering factor at 0.5 of B-site element
M_A_	Relative atomic mass of A-site element [55]
M_B_	Relative atomic mass of B-site element
DCE_A_	Core electron distance (Schubert) of A-site element [55]
DCE_B_	Core electron distance (Schubert) of B-site element
DVE_A_	Valence electron distance (Schubert) of A-site element [55]
DVE_B_	Valence electron distance (Schubert) of B-site element
AN_A_	Atomic number of A-site element in element period table
AN_B_	Atomic number of B-site element in element period table
E_A_-MB	A-site electronegativity-Matyonov-Batsanov [55]
E_B_-MB	B-site electronegativity-Matyonov-Batsanov
E_A_-P	A-site electronegativity-Pauling scale [55]
E_B_-P	B-site electronegativity-Pauling scale
EC_A_	A-site Energy cohesive (Brewer) [55]
EC_B_	B-site Energy cohesive (Brewer)
EI_A_	First energy ionization of A-site element [56]
EI_B_	First energy ionization of B-site element
SE_A_	A-site Solid entropy [55]
SE_B_	B-site Solid entropy
MT_A_	Melting point of A-site elements [55]
MT_B_	Melting point of B-site elements
NM_A_	A-site Nuclear magnetic moments [55]
NM_B_	B-site Nuclear magnetic moments
NEC_A_-C	Nuclear effective charge-Clementi of A-site element
NEC_B_-C	Nuclear effective charge-Clementi of B-site element
NEC_A_-S	Nuclear effective charge-Slater of A-site element [55]
NEC_B_-S	Nuclear effective charge-Slater of B-site element
QN_A_	A-site Quantum number [55]
QN_B_	B-site Quantum number
CR_A_	A-site Covalent radius [55]
CR_B_	B-site Covalent radius
PCR_A_	Pseudopotential core radii of A-site [57]
PCR_B_	Pseudopotential core radii of B-site
VEN/NC-A	Ratio of valence electron number to nuclear charge of A-site
VEN/NC-B	Ratio of valence electron number to nuclear charge of B-site
MV_A_	A-site Molar volume [55,56]
MV_B_	B-site Molar volume
AR_A_ (pm)	Atomic radius of A-site element [58]
AR_B_ (pm)	Atomic radius of B-site element
IR_A_ (Å)	Shannon’s (1976) ionic radii of A-site [59]
IR_B_ (Å)	Shannon’s (1976) ionic radii of B-site [59]
A-O (Å)	Ideal A-O bond distance [60]
B-O (Å)	Ideal B-O bond distance
EVR_A_	Equilibrium van der Waals radii of A-site element [61]
EVR_B_	Equilibrium van der Waals radii of B-site element
CVR_A_	Crystallographic van der Waals radii of A-site element [61]
CVR_B_	Crystallographic van der Waals radii of B-site element
EA_A_	Electron affinity of A-site element [56]
EA_B_	Electron affinity of B-site element
P_A_	Polarizability of A-site element [62,63]
P_B_	Polarizability of B-site element
EP_A_	Element period number of A-site element
EP_B_	Element period number of B-site element
C_A_-a (Å)	Cell parameters of A-site element in the a direction [56]
C_B_-a (Å)	Cell parameters of B-site element in the a direction
C_A_-b (Å)	Cell parameters of A-site element in the b direction [56]
C_B_-b (Å)	Cell parameters of B-site element in the b direction
C_A_-c (Å)	Cell parameters of A-site element in the c direction [56]
C_B_-c (Å)	Cell parameters of B-site element in the c direction
ID_A_	A-site Ionic displacement [64]
ID_B_	B-site Ionic displacement
t	Tolerance factor calculated by Shannon’s ionic radii
μ	Octahedral factor calculated by Shannon’s ionic radii
ST (°C)	Sintering temperature

## Appendix A

### Φ Separating A-Site from B-Site

**Highly Relevant Features**	**Low/No Correlation Features**
DCE_A_, DVE_A_, EC_A_, EI_A_, SE_A_, MT_A_, NM_A_, CR_A_, PCR_A_, AR_A_, IR_A_, A-O, EVR_A_, CVR_A_, EA_A_, P_A_, C_A_-a, C_A_-b, C_A_-c, SF_B_, DVE_B_, E_B_-MB, E_B_-P, EI_B_, QN_B_, CR_B_, PCR_B_, AR_B_, B-O, EVR_B_, CVR_B_, μ	ST, VEN/NC-A, NM_B_, EA_B_, t
SF_A_, M_A_, NEC_A_-C, AN_A_, E_A_-MB, E_A_-P, NEC_A_-S, QN_A_, EP_A_, ID_A_	
ID_B_, VEN/NC-B, IR_B_, P_B_, EP_B_	
DCE_B_, NEC_B_-C, SE_B_, NEC_B_-S, MV_B_, C_B_-a, C_B_-b, C_B_-c	
MV_A_, EC_B_, MT_B_	
M_B_, AN_B_	

## Appendix B

### Appendix B.1. Neural Network Hyperparameter Reference

Component/Parameter	Configuration/Value Range	
Number of Neurons	Input Layer	11
	Hidden Layer	128 × 256 × 128
	Output Layer	1
Activation Function	SiLU (Swish); Tanh; Relu; SELU	
Dropout Rate	0.1–0.2	
Number of Epochs	4000–20,000	
Loss Function	Gaussian NLL Loss; MSELoss; Kullback–Leibler Divergence; MAELoss; Smooth L1 Loss	
Optimizer	AdamW; SGD; Adam; SparseAdam	
MC Dropout	50–250	

### Appendix B.2. Key Code (ResNet)

class ResidualBlock(nn.Module):

     def __init__(self, input_dim, output_dim, dropout_rate = 0.2):

          super(ResidualBlock, self).__init__()

          self.linear1 = nn.Linear(input_dim, output_dim)

          self.bn1 = nn.BatchNorm1d(output_dim)

          self.relu = nn.ReLU()

          self.dropout = nn.Dropout(dropout_rate)

          self.linear2 = nn.Linear(output_dim, output_dim)

          self.bn2 = nn.BatchNorm1d(output_dim)

          if input_dim != output_dim:

               self.shortcut = nn.Sequential(

                    nn.Linear(input_dim, output_dim),

                    nn.BatchNorm1d(output_dim)

               )

          else:

               self.shortcut = nn.Identity()

     def forward(self, x):

          identity = self.shortcut(x)

          out = self.linear1(x)

          out = self.bn1(out)

          out = self.relu(out)

          out = self.dropout(out)

          out = self.linear2(out)

          out = self.bn2(out)

          out += identity # Residual Connection

          out = self.relu(out)

          return out

## References

[1] Ma B., Wu X., Zhao C., Lin C., Gao M., Sa B., Sun Z. (2023). An interpretable machine learning strategy for pursuing high piezoelectric coefficients in (K_0.5_Na_0.5_) NbO_3_-based ceramics. npj Comput. Mater..

[2] Absalon D., Ślesak B. (2010). The effects of changes in cadmium and lead air pollution on cancer incidence in children. Sci. Total Environ..

[3] Yuan X., Huo R., Pei Q., Zhao G., Li Y. (2025). Uncertainty quantification for the 3D half-space sound scattering problem of IGABEM based on the Catmull- Clark subdivision surfaces. Eng. Anal. Bound. Elem..

[4] Zhao G., Xuan J., Xu Y., Li Y. (2025). Fast multipole boundary element method with subdivision surface for acoustic analysis under seabed and sea surface reflection conditions. Phys. Fluids.

[5] Takatani T., Eguchi A., Yamamoto M., Sakurai K., Takatani R., Taniguchi Y., Nakayama S.F., Mori C., Kamijima M. (2022). Individual and mixed metal maternal blood concentrations in relation to birth size: An analysis of the Japan Environment and Children’s Study (JECS). Environ. Int..

[6] Wang J., Deng P., Wei X., Zhang X., Liu J., Huang Y., She J., Liu Y., Wan Y., Hu H. (2023). Hidden risks from potentially toxic metal(loid)s in paddy soils-rice and source apportionment using lead isotopes: A case study from China. Sci. Total Environ..

[7] Zhang Y., Zhu Q., Tian B., Duan C. (2024). New-Generation Ferroelectric AlScN Materials. Nano-Micro Lett..

[8] Rosso M., Maspero F., Esposito A., Afifi T.A., Riani M., Gattere G., Di Matteo A., Corigliano A., Ardito R. (2026). Characterization of AlN in MEMS: Synergistic use of dynamic testing, static profilometry, and an enhanced reduced-order model. Eur. J. Mech. A/Solids.

[9] Tao H., Wu H., Liu Y., Zhang Y., Wu J., Li F., Lyu X., Zhao C., Xiao D., Zhu J. (2019). Ultrahigh performance in fead-free piezoceramics utilizing a relaxor slush polar state with multiphase coexistence. J. Am. Chem. Soc..

[10] Xu Y., Wei Z., Pei Q., Li X., Li Y. (2025). FEM-BEM Analysis with subdivision surface for acoustic analysis of thin-shell structures under sea floor and sea level refection conditions. Ocean Eng..

[11] Shen J., Gu W., Zhao G., Liu C., Zhai C., Lian H. (2025). Isogeometric boundary element analysis ofsensitivity in TE-polarized electromagnetic scalttering from dielectric bodies. Eng. Anal. Bound. Elem..

[12] Jing R., Yu B., Ren S., Chen L., Chen H., Yao W. (2025). A novel isogeometric scaled coordinate transformation boundary element method for identifying steady-state surface heat sources. Comput. Methods Appl. Mech. Eng..

[13] Li Y., Xuan J., Liu C., Zhao G., Xu Y. (2026). Shallow-water acoustic analysis with an accelerated isogeometric boundary element approach. Appl. Ocean Res..

[14] Dong L., Zhang X., Yang Z., Shen L., Lu Y. (2025). Accurate piezoelectric tensor prediction with equivariant attention tensor graph neural network. npj Comput. Mater..

[15] Artetxe E., Barambones O., Calvo I., del Rio A., Uralde J. (2024). Combined Control for a Piezoelectric Actuator Using a Feed-Forward Neural Network and Feedback Integral Fast Terminal Sliding Mode Control. Micromachines.

[16] Luo X., Wang Z., Gao P., Lv J., Wang Y., Chen C., Ma Y. (2024). Deep learning generative model for crystal structure prediction. npj Comput. Mater..

[17] Zhao W., Zhou M., Shao J., Ren J., Hu Y., Han Y., Man Y. (2025). Crystal Structure Prediction Using a Self-Attention Neural Network and Semantic Segmentation. J. Chem. Inf. Model..

[18] Sriboriboon P., Qiao H., Kwon O., Vasudevan R.K., Jesse S., Kim Y. (2023). Deep learning for exploring ultra-thin ferroelectrics with highly improved sensitivity of piezoresponse force microscopy. npj Comput. Mater..

[19] Zhang R., Motes B., Tan S., Lu Y., Shih M.-C., Hao Y., Yang K., Srinivasan S., Bawendi M.G., Bulović V. (2025). Machine Learning Prediction of Organic–Inorganic Halide Perovskite Solar Cell Performance from Optical Properties. ACS Energy Lett..

[20] Zhu W., Wang X., Gao W. (2020). Multimedia intelligence: When multimedia meets artificial intelligence. IEEE Trans. Multimed..

[21] Lian H., Li S., Li X., Xu Y., Chen L., Natarajan S. (2026). Underwater acoustic simulation from muli-view sonar images: A neus-assisted boundary element approach. Thin-Walled Struct..

[22] Lian H., Zhang Y., Bian N., Qu Y., Li Y., Chen L. (2025). Integrating 3dgs novel view synthesis and CFD for modeling bionic robotic fish from multi view imagery. Ocean Eng..

[23] Chen L., Lian H., Liu C., Li Y., Natarajan S. (2025). Sensitivity analysis of transverse electric polarized electromagnetic scattering withsogeometric boundary elements accelerated by a fast multipole method. Appl. Math. Model..

[24] Chen L., Liu C., Lian H., Gu W. (2025). Electromagnetic scattering sensitivity analysis for perfectly conducting objects in tm polarization with isogeometric BEM. Eng. Anal. Bound. Elem..

[25] Samek W., Montavon G., Vedaldi A., Hansen L.K., Müller K.R. (2019). Explainable AI: Interpreting, Explaining and Visualizing Deep Learning.

[26] Chen L., Pei Q., Fei Z., Zhou Z., Hu Z. (2025). Deep-neural-network based framework for the accelerating uncertainty quantification of astructural-acoustic fully coupled system in a shallow sea. Eng. Anal. Bound. Elem..

[27] Chen L., Huo R., Lian H., Yu B., Zhang M., Natarajan S., Bordas S.P. (2025). Uncertainty quantification of 3d acoustic shape sensitivities with generalized nth-order perturbation boundary element methods. Comput. Methods Appl. Mech. Eng..

[28] Saleem R., Yuan B., Kurugollu F., Anjum A., Liu L. (2022). Explaining deep neural networks: A survey on the global interpretation methods. Neurocomputing.

[29] Adadi A., Berrada M. (2018). Peeking inside the black-box: A survey on explainable artificial intelligence (XAI). IEEE Access.

[30] Das A., Rad P. (2020). Opportunities and Challenges in Explainable Artificial Intelligence (XAI): A Survey. arXiv.

[31] Tjoa E., Guan C. (2020). A survey on explainable artificial intelligence (XAI): Toward medical XAI. IEEE Trans. Neural Netw. Learn. Syst..

[32] Erhan D., Bengio Y., Courville A., Vincent P. (2009). Visualizing higher-layer features of a deep network. Univ. Montr..

[33] Nguyen A., Dosovitskiy A., Yosinski J., Brox T., Clune J. (2016). Synthesizing the preferred inputs for neurons in neural networks via deep generator networks. Adv. Neural Inf. Process. Syst..

[34] Bykov K., Deb M., Grinwald D., Muller K.R., Höhne M.M.C. (2022). DORA: Exploring outlier representations in deep neural networks. Trans. Mach. Learn. Res..

[35] Bykov K., Kopf L., Nakajima S., Kloft M., Höhne M.M.-C. (2023). Labeling neural representations with inverse recognition. arXiv.

[36] Bau D., Zhou B., Khosla A., Oliva A., Torralba A. Network dissection: Quantifying interpretability of deep visual representations. Proceedings of the IEEE Conference on Computer Vision and Pattern Recognition.

[37] Mu J., Andreas J. (2020). Compositional explanations of neurons. Adv. Neural Inf. Process. Syst..

[38] Dombrowski A.-K., Anders C.J., Müller K.-R., Kessel P. (2021). Towards robust explanations for deep neural networks. Pattern Recognit..

[39] Lundberg S.M., Erion G., Chen H., DeGrave A., Prutkin J.M., Nair B., Katz R., Himmelfarb J., Bansal N., Lee S.-I. (2020). From local explanations to global understanding with explainable ai for trees. Nat. Mach. Intell..

[40] Xu G., Duong T.D., Li Q., Liu S., Wang X. (2020). Causality learning: A new perspective for interpretable machine learning. arXiv.

[41] Yuan R., Liu Z., Balachandran P.V., Xue D., Zhou Y., Ding X., Sun J., Xue D., Lookman T. (2018). Accelerated discovery of large electrostrains in BaTiO_3_-based piezoelectrics using active learning. Adv. Mater..

[42] He J., Yu C., Hou Y., Su X., Li J., Liu C., Xue D., Cao J., Su Y., Qiao L. (2022). Accelerated discovery of high-performance piezo catalyst in BaTiO_3_-based ceramics via machine learning. Nano Energy.

[43] He J., Li J., Liu C., Wang C., Zhang Y., Wen C., Xue D., Cao J., Su Y., Qiao L. (2021). Machine learning identified materials descriptors for ferroelectricity. Acta Mater..

[44] Yuan R., Tian Y., Xue D., Xue D., Zhou Y., Ding X., Sun J., Lookman T. (2019). Accelerated search for BaTiO_3_-based ceramics with large energy storage at low fields using machine learning and experimental design. Adv. Sci..

[45] Yuan R., Xue D., Xue D., Li J., Ding X., Sun J., Lookman T. (2020). A knowledge-based descriptor for the compositional dependence of the phase transition in BaTiO3-based ferroelectrics. ACS Appl. Mater. Interfaces.

[46] Yuan R., Xue D., Xu Y., Xue D., Li J. (2022). Machine learning combined with feature engineering to search for BaTiO_3_ based ceramics with large piezoelectric constant. J. Alloy Compd..

[47] Ouyang R., Curtarolo S., Ahmetcik E., Scheffler M., Ghiringhelli L.M. (2018). SISSO: A compressed-sensing method for identifying the best low-dimensional descriptor in an immensity of offered candidates. Phys. Rev. Mater..

[48] Bartel C.J., Sutton C., Goldsmith B.R., Ouyang R., Musgrave C.B., Ghiringhelli L.M., Scheffler M. (2019). New tolerance factor to predict the stability of perovskite oxides and halides. Sci. Adv..

[49] Oh S.-H.V., Hwang W., Kim K., Lee J.-H., Soon A. (2022). Using feature-assisted machine learning algorithms to boost polarity in lead-free multicomponent niobate alloys for high-performance ferroelectrics. Adv. Sci..

[50] Box G.E.P., Cox D.R. (1964). An Analysis of Transformations. J. R. Stat. Soc. Ser. B Methodol..

[51] Chai Y., Liu Y., Li W., Zhu B., Liu H., Jiang Y. (2024). An interpretable wide and deep model for online disinformation detection. Expert Syst. Appl..

[52] Guo H., Tang R., Ye Y., Li Z., He X. (2017). DeepFM: A Factorization-Machine based Neural Network for CTR Prediction. arXiv.

[53] Chen D., Hu F., Nian G., Yang T. (2020). Deep Residual Learning for Nonlinear Regression. Entropy.

[54] Aghaei A., Moghaddam M.E., Initiative A.D.N. (2024). Brain age gap estimation using attention-based ResNet method for Alzheimer’s disease detection. Brain Inform..

[55] Stolyarenko M.A. (2023). Russian Academy of Sciences A.A. Baikov Institute of Metallurgy and Materials Science Database on Properties of Chemical Elements. https://phases.imet-db.ru/elements/main.aspx.

[56] Winter M.J. (2015). WebElements. https://www.webelements.com.

[57] Zunger A. (1980). Systematization of the stable crystal structure of all AB-type binary compounds: A pseudopotential orbital-radii approach. Phys. Rev. B.

[58] Samsonov G.V. (1968). Handbook of the Physicochemical Properties of the Elements.

[59] Shannon R.D. (1976). Revised effective ionic radii and systematic studies of interatomic distances in halides and chalcogenides. Acta Crystallogr..

[60] Brese N.E., O’keeffe M. (1991). Bond-valence parameters for solids. Acta Crystallogr..

[61] Batsanov S.S. (2001). Van der waals radii of elements. Inorg. Mater..

[62] You X. (1974). Ionic polarizability. Chin. Sci. Bull..

[63] Feng Y., Zhang S., Sun H., Li Y., Zhun Y. (2000). Ionic polarizability. J. Dalian Inst. Light Ind..

[64] Grinberg I., Rappe A.M. (2007). First principles calculations, crystal chemistry and properties of ferroelectric perovskites. Phase Transit..

[65] Grinberg I., Rappe A.M. (2004). Local structure and macroscopic properties in PbMg_1/3_Nb_2/3_O_3_-PbTiO_3_ and PbZn_1/3_Nb_2/3_O_3_-PbTiO_3_ solid solutions. Phys. Rev. B.

[66] Zhang N., Zheng T., Wu J. (2020). Lead-free (K,Na)NbO_3_-based materials: Preparation techniques and piezoelectricity. ACS Omega.

[67] Cen Z., Wang X., Huan Y., Li L. (2018). Temperature stability and electrical properties of MnO-doped KNN-based ceramics sintered in reducing atmosphere. J. Am. Ceram. Soc..

[68] Cen Z., Wang X., Huan Y., Zhen Y., Feng W., Li L. (2018). Defect engineering on phase structure and temperature stability of KNN-based ceramics sintered in different atmospheres. J. Am. Ceram. Soc..

